# Effective transcription factor binding site prediction using a combination of optimization, a genetic algorithm and discriminant analysis to capture distant interactions

**DOI:** 10.1186/1471-2105-8-481

**Published:** 2007-12-19

**Authors:** Victor G Levitsky, Elena V Ignatieva, Elena A Ananko, Igor I Turnaev, Tatyana I Merkulova, Nikolay A Kolchanov, TC Hodgman

**Affiliations:** 1 Institute of Cytology and Genetics SB RAS, Novosibirsk, 630090, Russia; 2 Novosibirsk State University, Novosibirsk, 630090, Russia; 3 Multidisciplinary Centre for Integrative Biology, School of Biosciences, University of Nottingham, Sutton Bonington, LE12 5RD, UK

## Abstract

**Background:**

Reliable transcription factor binding site (TFBS) prediction methods are essential for computer annotation of large amount of genome sequence data. However, current methods to predict TFBSs are hampered by the high false-positive rates that occur when only sequence conservation at the core binding-sites is considered.

**Results:**

To improve this situation, we have quantified the performance of several Position Weight Matrix (PWM) algorithms, using exhaustive approaches to find their optimal length and position. We applied these approaches to bio-medically important TFBSs involved in the regulation of cell growth and proliferation as well as in inflammatory, immune, and antiviral responses (NF-κB, ISGF3, IRF1, STAT1), obesity and lipid metabolism (PPAR, SREBP, HNF4), regulation of the steroidogenic (SF-1) and cell cycle (E2F) genes expression. We have also gained extra specificity using a method, entitled SiteGA, which takes into account structural interactions within TFBS core and flanking regions, using a genetic algorithm (GA) with a discriminant function of locally positioned dinucleotide (LPD) frequencies.

To ensure a higher confidence in our approach, we applied resampling-jackknife and bootstrap tests for the comparison, it appears that, optimized PWM and SiteGA have shown similar recognition performances. Then we applied SiteGA and optimized PWMs (both separately and together) to sequences in the Eukaryotic Promoter Database (EPD). The resulting SiteGA recognition models can now be used to search sequences for BSs using the web tool, SiteGA.

Analysis of dependencies between close and distant LPDs revealed by SiteGA models has shown that the most significant correlations are between close LPDs, and are generally located in the core (footprint) region. A greater number of less significant correlations are mainly between distant LPDs, which spanned both core and flanking regions. When SiteGA and optimized PWM models were applied together, this substantially reduced false positives at least at higher stringencies.

**Conclusion:**

Based on this analysis, SiteGA adds substantial specificity even to optimized PWMs and may be considered for large-scale genome analysis. It adds to the range of techniques available for TFBS prediction, and EPD analysis has led to a list of genes which appear to be regulated by the above TFs.

## Background

Transcription factors (TFs) function by binding to the recognition sites in gene regulatory regions. TF binding to DNA is mediated through base-dependent hydrogen bonds and other structural propensities that are the result of dinucleotide stacking: salt bridges to the phosphate backbone, Hydrogen bonds and Van der Waal's interactions [1]. TFs are also often members of multimolecular complexes to which the DNA binds through further sequence and structural features. Where TFBS sequences are known, one can try to search for similar sequences computationally. These binding sites are often represented by a consensus, which is just a pattern of bases that occur at specific positions in a site. Because the sites are often degenerate, mismatches to the consensus are often admissible. But consensus presentation has limited use for even moderately variable BSs, because it preserves too little or no information about nucleotide variability.

TFBS prediction is usually attempted using position weight matrices (PWMs) [2-4]. This method implies that there are some contributions from each base at each position and that the sum of all these contributions is above a certain threshold. However, this is inadequate for three reasons. The core sequence-specific positions are so few that the matrices have a high false positive rate. Many TFBSs have too few functionally characterised sequences to populate, to a statistically meaningful extent, a dinucleotide PWM of sufficient length to capture the long-range structural propensities. Finally, they are severely limited by the assumption that positions in a site contribute additively to the total score. Experimental evidence suggests that this assumption of independence is not always true [5-7]. This assumption may be just a good and useful approximation, which however does not fit data perfectly and is therefore not quite correct [8].

The high false-positive rates in TFBSs prediction using PWMs of the core motifs have led to various attempts to draw in extra information to improve performance [9,10]. One is to look for conservation of predicted BSs between homologous genes of different species taking into account the evolutionary distances [11,12], though this will result in the potential elimination of species-specific TFBSs. Given that TFs usually act through multi-protein complexes, another has been to search for pairs or higher multiples of TFBSs [13]. This approach is restricted either to known or presumed TFBS pairs.

The simplest way to increase performance of conventional PWM is the calculation of dependencies between adjacent positions. This model is represented by a dinucleotide PWMs (or weight array model) [14,15]. Besides the obvious advantage of involving higher-order statistics, they certainly may capture the longest lengths of motifs. Another tool for TFBSs prediction used PWMs constructed on the basis of degenerate oligonucleotide motifs [16]. This approach may represent more than two non-adjacent positions together, but still the motif structure is preliminary restricted. Additional statistical features in the flanking regions of sites may also support TFBS recognition [17]. This analysis uses many types of features, for instance oligonucleotide content, structural and chemical context-dependent parameters like helical twist or melting temperature. A similar approach, based on discriminant function of retrieved features to E2F BSs, appears to be very promising [18]. Namely, the false positive rate of PWM may be substantially decreased and this especially refers to revealing high scoring sites.

Another successful approach for PWM improvement incorporated position-dependent information content and pairwise correlations [19]. In this work the notion of scope delimited the correlating nucleotides (e.g. a scope of two considers both adjacent and separated by an intermediate nucleotide pairs). Recently, other approaches have been reported for PWM improvement by the consideration of dependencies between distant positions [20-25].

The interaction of distant site positions can be important for the formation of DNA secondary structure that aids TF recruitment, its interaction with DNA duplex and stable TF-DNA complex formation. Therefore correlations between arbitrary BS positions are expected to be important. These correlations provide long-range structural rather than sequence-specific interactions. Some TFs function through association with nucleosomes [26-29], which do not bind to any sequence-specific motif but rather through quaternary structures of the duplex DNA [30].

In this work we have further developed a new method, entitled SiteGA [31], using a genetic algorithm (GA) involving a discriminant function of locally positioned dinucleotides (LPDs) and applied it to clinically important TFBSs. It is derived from the analysis of local dinucleotide context [32,33], and provides the subtlety of discriminant analysis [34-36,18] with the speed of a GA in detecting significant features. To evaluate the performance of our approach, we compared it with optimized PWMs, whose lengths were adjusted until they performed at their best and also compared mono- and dinucleotide matrices.

Duplex-DNA quaternary structures result from the DNA bending and flexibility, which arise from the stacking interactions of successive dinucleotides [37]. Such structural approaches could be defined by a dinucleotide PWM, but this would require hundreds of sites to develop a statistically meaningful 16 × (*L*-1) matrix (where *L *denotes site length). Discriminant analysis provides an approach to determining which dinucleotides and positions appear to be significant.

The pattern beyond the canonical footprint reflects the genome nucleotide context of neighbour regulatory regions around BSs. Consideration of these regions helps to increase the recognition accuracy. Subtle context features besides the site footprint may be related to the presence of other still unknown features within the overall regulatory element (for example other TFBSs). Finally we combined the SiteGA and optimized PWM models together and applied them to the human promoters from EPD [38]. This reveals the most reliable potential BS targets.

## Results

### PWMs: window lengths and performance

**Figure 1:**
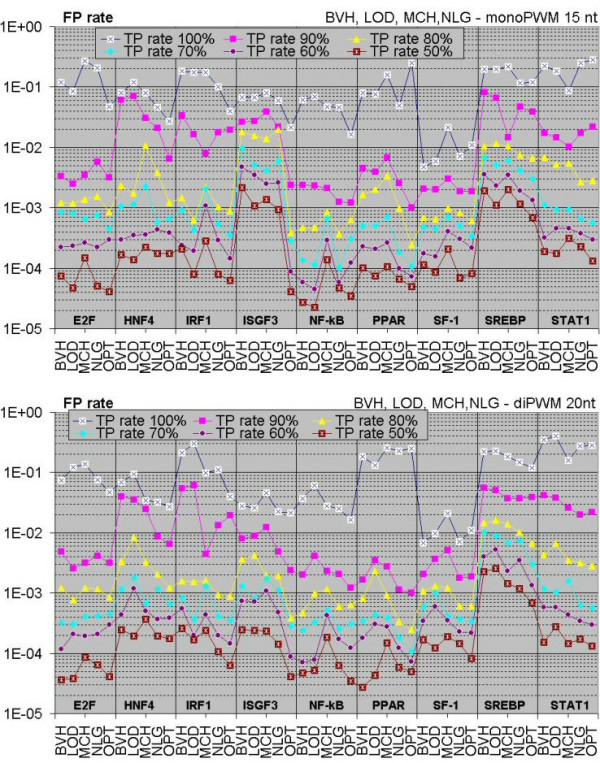
**Recognition performance for different PWMs**. Each vertical block shows the performance of five different PWM algorithms: BVH (Berg and von Hippel) [2], LOD (log-odds) [63], MCH (MATCH) [62], NLG (natural logarithm) and OPT (natural logarithm, optimized matrix length) for each TF. The vertical axis shows the false-positive rate (logarithmic scale) for that algorithm at true-positive rates defined in the caption at the top of the figure. The upper and lower plots compare the algorithms using 15 nt mononucleotide and 20 nt dinucleotide PWMs respectively.

Figure 1 depicts modified receiver operating characteristic (ROC) plots computed by jackknife resampling tests [39] for different PWM models of 9 TFBSs (E2F, NF-κB, ISGF3, IRF1, PPAR, SF-1, HNF4, SREBP and STAT1). At any given true positive (TP) rate, better performance is indicated by a lower false positive (FP) value. The general impression is that the NLG algorithm (developed here) performs at least as well as and often better than the other algorithms, with optimized matrices doing better still. This is substantiated by average rankings for mono- and diPWM shown in Table 1 and Table 2, correspondingly.

**Table 1:** Average ranking of 5 monoPWM models (BVH, LOD, MCH, NLG and optimized NLG (OPT)) calculated for corresponding FP rates (matrix length 15 nt).

TP rate, %	BVH	LOD	MCH	NLG	OPT
50	3.78	2.00	4.78	2.78	1.67
60	3.11	2.44	4.67	2.44	2.33
70	4.11	2.89	4.22	2.22	1.56
80	3.11	2.78	4.56	3.00	1.56
90	4.11	3.00	2.89	3.00	2.00
100	3.44	3.22	3.67	2.44	2.22

The average ranks calculated on the basis of nine TFBS types (see methods, Sequence data)

**Table 2:** Average ranking of 5 diPWM models (BVH, LOD, MCH, NLG and optimized NLG (OPT)) calculated for corresponding FP rates (matrix length 20 nt).

TP rate, %	BVH	LOD	MCH	NLG	OPT
50	3.44	3.00	4.22	3.00	1.33
60	3.11	3.78	3.89	2.44	1.67
70	3.78	3.44	4.11	2.22	1.44
80	3.56	4.22	3.89	2.00	1.33
90	3.89	4.33	3.00	2.00	1.78
100	3.11	3.89	3.67	2.44	1.89

The average ranks calculated on the basis of nine TFBS types (see methods, Sequence data)

**Figure 2:**
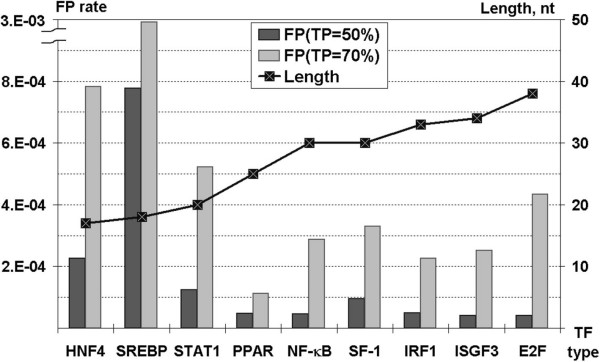
**FP rates for the optimized matrices**. The FP rates for each optimized PWM are plotted for 50% and 70% TP rates. The PWMs have also been arranged from left to right in order of sequence length, with the length axis provided on the right-hand side of the plot.

Detailed values for the optimized PWM models are in Table 3, with Figure 2 showing FP rates for the optimized models according to model length. We found that motif lengths should be greater than 20 bases long for lower FP rates, and generally that dinucleotide slightly outperformed mononucleotide PWMs, for all except SREBP. One might have expected that the structural information implicit in dinucleotide stacks would have resulted in a generally much better performance. We attribute this meagre improvement to the shortage of sequences available to populate a 16 × (*L *- 1) matrix needed for dinucleotides (where *L *denotes site length).

**Table 3:** Details for PWM and SiteGA models of TFBS recognition

	General parameters		Specific parameters	
	
TF type	No. of training sequences	Window length, nt	PWM Type of matrix^1^	SiteGA Number of LPDs
E2F	40	38	DI	60
HNF4	29	17	DI	140
IRF1	28	33	DI	38
ISGF3	27	34	DI	36
NF-κB	43	30	DI	150
PPAR	37	25	DI	90
SF-1	53	30	DI	90
SREBP	37	18	MONO	110
STAT1	32	20	DI	150

^1 ^– DI – dinucleotide PWM, MONO – mononucleotide PWM.

### PWMs and SiteGA: performance comparison

**Figure 3:**
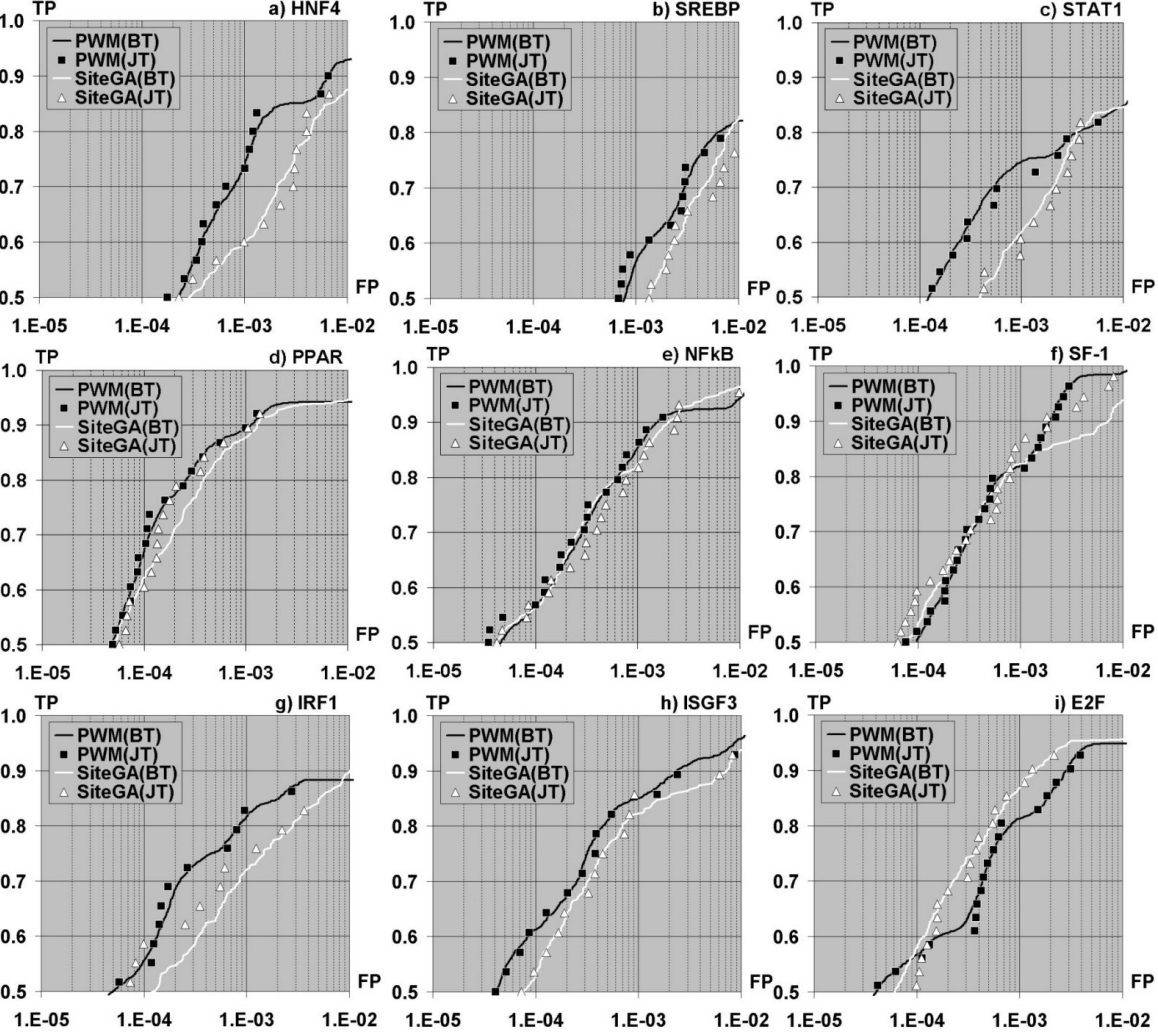
**ROC plots of SiteGA and PWM models**. The plots compare performance of the two approaches for the 50–80% TP region. a) HNF4, b) SREBP, c) STAT1, d) PPAR, e) NF-κB, f) SF-1, g) IRF1, h) ISGF3, i) E2F. In this instance, better performance is marked by higher values and plots positioned further to the left. FP rates (X axis) are in logarithmic scale.

SiteGA represents a radically different approach to PWMs in that the Genetic Algorithm has discovered the dinucleotide interactions that are most significant, with probabilities assigned to their ability to discriminate between genuine and false sites. The significant dinucleotide pairs may be very far apart, and the intrinsic information not detectable using PWMs. Its parameters for the nine TFBSs are also shown in Table 3, and Figure 3 shows the ROC plots for SiteGA and PWM models.

The most striking feature of these results is that the SiteGA approach often performed close to and sometimes better than the optimized PWMs. For the latter, the jackknife versus the bootstrap techniques gave closely similar plots, whereas for the former, there was a wider disparity between the two. It might be an artefact of the procedure, but the jackknife technique tended to give a step-wise series of steeper curves. The plots have been ordered according to sequence length and it is clear that the shorter sites (HNF4, SREBP, and STAT1) performed the least well by either method, i.e. at 50% TP rates, the FP rate was >1.E-04. Of the remainder, SiteGA did not perform as well as the optimized PWMs for IRF1 and ISGF3, but this can be accounted for by the comparatively low number of sites in the set (28 and 27 respectively) leading to the low number of discriminatory LPDs (38 and 36 respectively). Larger datasets could improve this performance. By contrast, SiteGA outperforms PWMs at most TP rates for E2F, which has the longest sequence length and the third highest number of representative sequences.

### SiteGA: patterns beyond the canonical core sequences

**Figure 4:**
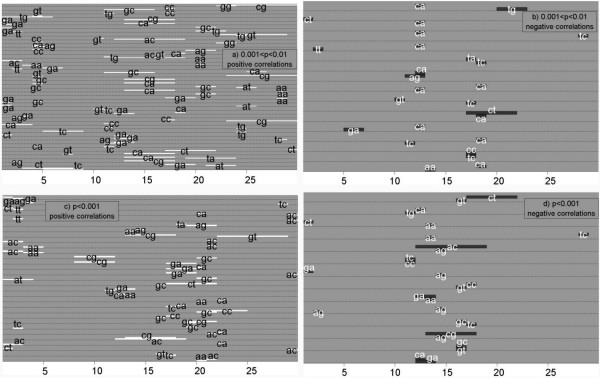
**Significant correlations between frequencies of locally positioned dinucleotides (LPDs) for SF-1 BSs**. Each horizontal strip depicts one correlation between two LPDs. a) Positive correlations, 0.001 < p < 0.01; b) negative correlations, 0.001 < p < 0.01; c) positive correlations, p < 0.001; d) negative correlations, p < 0.001. Whole analyzed 30 bp long region corresponds to [1;30] window of 29 dinucleotide positions. Also note that the SF-1 consensus sequence GTCAAGGTCA [39] located within region [10;19].

One of the most interesting questions in our study was to clarify the nature of context patterns both within and outside of the well-known site core region. For this we looked in detail at SF-1, for which we have the largest dataset (see Figure 4). We found that the most significant context features were inherent to the consensus [10;19] and footprint (approximately [5;25]) regions. Locations of dinucleotides were defined with respect to dinucleotide positions. For example, the second bottom pair of LPDs in Figure 4c show a 'positive correlation between [16;16] GT and [17;17] TC'. It means mutual occurrence of dinucleotides GT and TC at 16 and 17 positions correspondingly. This positive correlation means that there is a frequent occurrence of trinucleotide GTC spanning nucleotides16–18. Indeed, it belongs to the consensus sequences gtcaagGTCa [40].

The first bottom pair of LPDs is the negative correlation between [12;13] GA and [12;12] CA. This means that when CA is found commencing at position 12, GA is never found at position 12 and rarely if ever found at position 13. Furthermore, the second bottom line in Figure 4d presents the negative correlation between dinucleotide GC and GT resided to the coincident location [16;16]. This corresponds to 50 out of 53 sites having C or T and position 17.

Note that the locations of regions corresponding to significant correlations are not restricted to the core consensus region. In general, the majority of the most significant correlations (p < 0.001) located within consensus region [10;19], less significant correlations (0.001 < p < 0.05) covered both consensus and flanking regions ([1;9] and [20;30]). There are also more positive rather than negative correlations, perhaps indicative of co-operative structural binding propensities.

**Figure 5:**
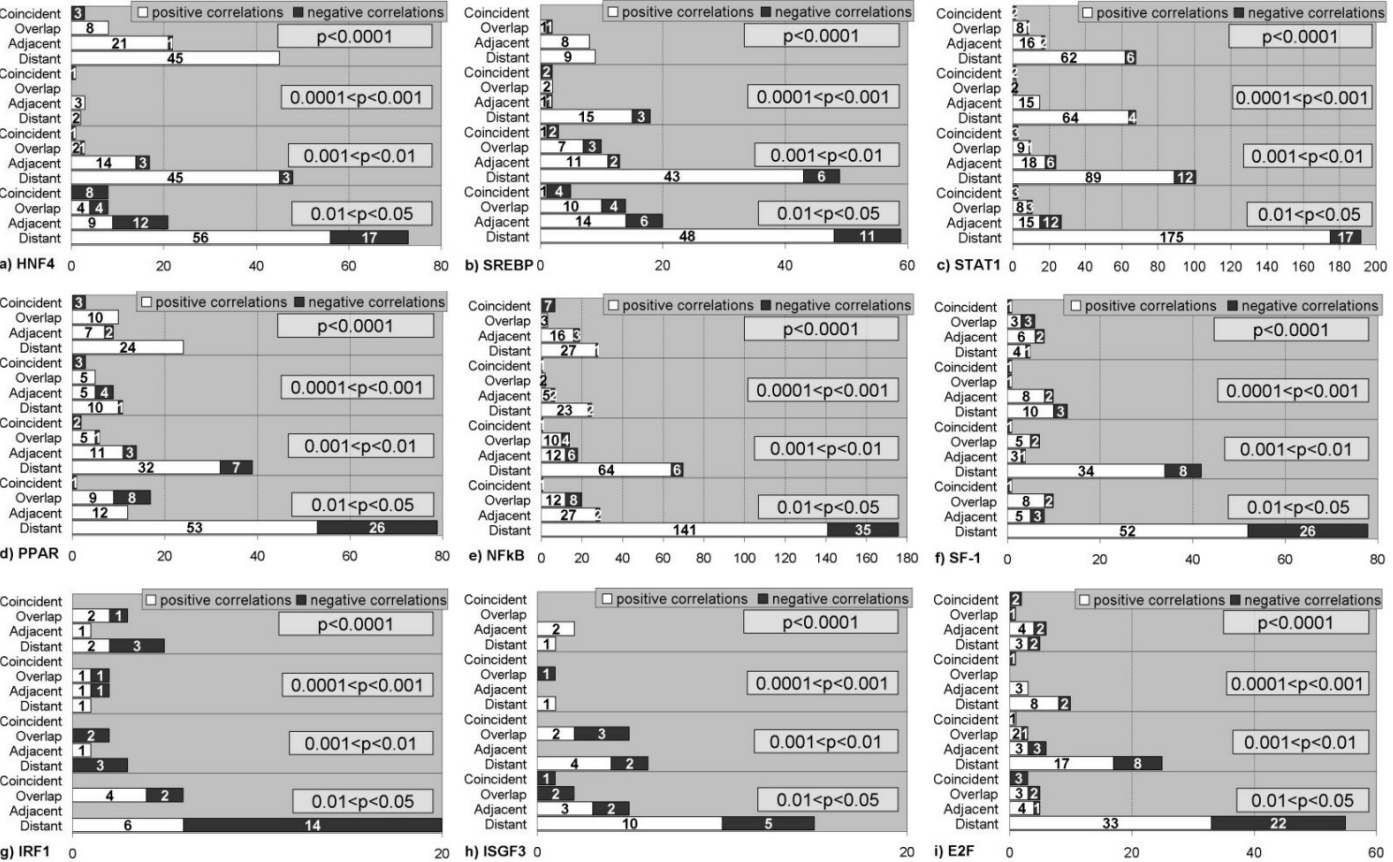
**Analysis of significant correlations (p < 0.05) between locally positioned dinucleotides frequencies calculated for SiteGA models**. a) HNF4, b) SREBP, c) STAT1, d) PPAR, e) NF-κB, f) SF-1, g) IRF1, h) ISGF3, i) E2F.

Figure 5 shows the analysis of significant correlations between LPDs for all the SiteGA models. Firstly, we separately considered close and distant dependent dinucleotides, then we partitioned close locations into coincident, overlapping and adjacent. Namely, for locations [*a*_1_; *b*_1_] and [*a*_2_; *b*_2_] of two LPDs only four types of mutual location were possible: (1) coincident, if *a*_1_*= a*_2 _and *b*_1_*= b*_2_; (2) overlapping, if *a*_1 _<*a*_2 _<*b*_1 _or *a*_1 _<*b*_2 _<*b*_1 _or *a*_2 _<*a*_1 _<*b*_2 _or *a*_2 _<*b*_1 _<*b*_2_; (3) adjacent, if *b*_1_*+ *1= *a*_2 _or *b*_2_*+ *1 = *a*_1_, (4) distant – in all remaining cases, i.e. *b*_1_*+*1 <*a*_2 _or *b*_2_*+*1 <*a*_1_. Generally, we found comparable portions of distant and close (sum of coincident, overlapping and adjacent) dependencies among the more significant correlations (p < 0.001). In some cases, the number of close dependencies was larger than number of distant ones. The opposite trend was observed for the less significant correlations (0.001 < p < 0.05). Namely, the distant correlations are prevailing over the close ones.

### PWMs, SiteGA and their combination applied for EPD promoter analysis

**Figure 6:**
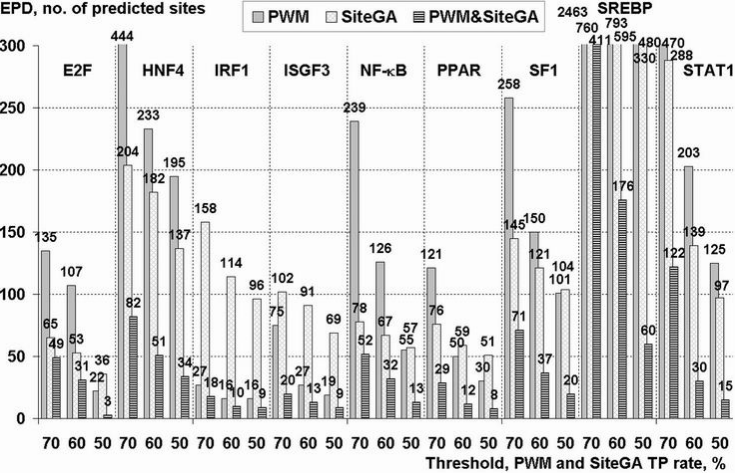
**Analysis of TFBSs predictions in EPD promoters by SiteGA, PWMs and combined PWM & SiteGA approach**. X-axis – TF types and stringencies in terms of fixed TP rates (50, 60 and 70% for SiteGA and PWMs); Y-axis – no. of predicted sites (data labels are marked for each point). Combined approach implied that both PWM and SiteGA models recognized a potential site.

Figure 6 shows the results of searching the Eukaryotic Promoter Database (EPD) with optimized PWMs, SiteGA and the two together. To make the evaluation more straightforward to interpret, the numbers of potential sites were counted for only three stringencies, corresponding to 50, 60 and 70% TPs calculated during the training and testing above. At lower stringencies, predicted sites were found more than once in a given promoter sequence, but these cases were very seldom.

Without exception, for a given TF and search method, the number of sites predicted increased with higher TP rates. This is as expected, since a greater number of TPs will add an increasing proportion of FPs, and, as a crude guide, higher numbers correspond to greater numbers of FPs being found. As expected from observations above, HNF4, SREBP and STAT1 predicted many sites, which almost certainly reflect the weak consensus leading to a higher proportion of FPs. In these cases, SiteGA consistently found fewer sites, perhaps indicative of better performance. IRF1, ISGF3 and to a lesser extent PPAR had smaller training sets, which resulted in fewer sequences retrieved from EPD, though SiteGA found more than PWMs probably because the former models were still poorly defined. For the remainder (E2F, NF-κB and SF1), PWMs returned fewer sequences at the highest stringency (which might reflect a genuine failure to identify functional sites) but more at the lower stringencies (more indicative of a higher FP rate).

**Figure 7:**
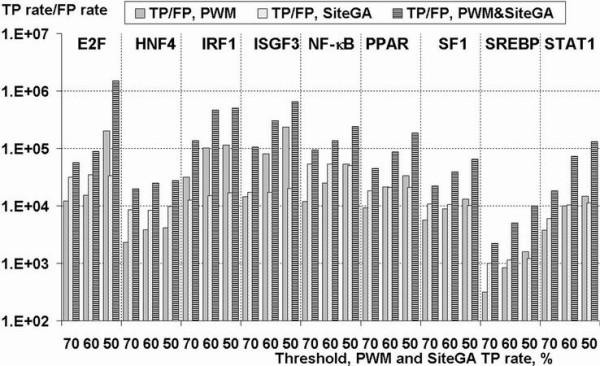
**Ratios between true positive (TP) and false positive (FP) rates calculated respectively on the basis of training and background data for SiteGA, PWMs and combined PWM & SiteGA approach**. X-axis – TF types and stringencies in terms of fixed TP rate (50, 60 and 70% for SiteGA and PWMs); Y-axis (logarithmic scale) – ratios of frequencies of predictions for the train (TP) to those for background set (FP). Combined approach implied that both PWM and SiteGA models recognized a potential site.

The combined search approach returned still fewer sequences, indicative of this being the most stringent approach and any given stringency. In order to confirm that this refinement was a consequence of discounting FPs, rather than just a proportional reduction of TPs and FPs, we computed the ratios TP/FP for all TFBSs for SiteGA, PWM and the combined approach (Figure 7). TP rates were estimated on the basis actual recognition rates for the training data and FP rates were evaluated as actual frequencies of predictions for background shuffled training sequences.

These calculations finally confirmed that:

(1) SiteGA models generally have higher ratios TP/FP than PWMs, indicating that the former may discriminate better between true and false sites;

(2) For any TFBSs TP/FP ratios are considerably larger for combined approach than for any separate model, thus the combination indeed is superior with respect to any single models.

The EPD genes retrieved by the combined approach are presented in Table 4. NF-κB was excluded from this study because the detected genes had a broad range functional roles that appeared contradictory [41], while SREBP was discounted for similar reasons and because it might still have a high number of FPs. Predicted sites for the remaining seven TFBSs were sorted by several criteria. Apart from the genes known and strongly suspected of being regulated by the TFs, the "possibly regulated" column includes genes that might be part of the physiological response mediated by the TF. The unrelated and unknown genes might also, but we have no evidence to support their involvement. Some TFs belong to gene families whose binding sites are similar, these genes might be regulated by the TF in question, or their identification might be the accidental result of sequence similarity.

**Table 4:** Ranking of EPD human promoters containing predicted TFBSs by target functionality

TF type, Stringency	Known targets	Very possible targets	Possible targets	BSs of homologous TFs	Possibly unrelated	Unknown
E2F, 60%	MCM7, MCM5, MCM2, Cyclin D1, RAD51, TYMS^2^	MCM3	SLC1A5, ALDOA E3P2, GORASP2, BTG1, RAE1, ZNF9, CDC37		MYL6, COX7B, COX8, YME1L1, MFGE8, TPMT	CBARA1, AUP1, SFRS10, PGD, TARS, PRPS1, RNASE4, PAPOLA^2^, MRPS7
HNF4, 50%	hepatic lipase	apolipop. B, apolipop. A1, glucagon, HPD, GLUL	HGD, HBXIP, CSTB, Cyclin D1, CCNB1, CCNB2, UBL5, TAPBP, IFI27, NDUFA6		histone H4-A1, PRM2, STIP1, RPS8, CASQ2, TOMM22, AUP1, SNX6, TRAP1, RPL23	CEA, FLJ13154, FLJ10276, LOC57862, COPS4, BLCAP, SLC22A8
IRF1, 80%	complement f. B, b'-interferon, HLA C, HLA B, IFI27, SP100, IFIT1, IFI 54K, IFI 6–16^2^, ISG 15K	BST2, B2M	Haptoglob Hp1F, haptoglob HpR, IL-4 (BSF-1), TAPBP, NDUFC2, PHGDH, GLRX^2^,	† CSF-1, PCNA		APOL3, SRP54, GTF2H4, DNASE2, NDUFB4
ISGF3, 70%	IFI27^2^, IFI 54K, IFI 6–16^2^, ISG 15K	Complement f. B, IFIT1	SSBP1, ATP5J, SCARB2, ESRRBL1, CTNNBL1, STX10, PMAIP1		TNP1, B4GALT4, KNS2, PPP1CA,	MGC2714
PPAR, 70%	ATIII	apolipop. CIII, PCK2	SDHD, CIR, LOC51064		histone H2B, BAP29, CHEK1, HNMT, HLA DPB1, MT-IB, MRPL11, SERPINA3, SGT, TNP1, TAPBP, TCL1A, UBL5, VPS29, ARAF1	CDW52, CSNK1A1, KIAA0971, PCBP2, STIP1, TAF9, TM4SF2, TMP21
SF-1, 60%	CG/LH/FSH/TSH-a, CYP11A, HSD3B2	ACPP, PSMD8	gastrin, FXYD3, CKMT2, MYL2, TGFB1, FXR1, VAPA, TPI1, CDC42EP2, MRPL11, MRPS21, S100A2	# CTRB1, PPGB, ATF4	SAT, CNTF, PPA1^2^, RPLP1, FARSL, SERPIND1, CASQ2, TXN, CCT7, SNRP70	ATP6V0D1, IGF II E3P3, ERH, OS, HU, JM5
STAT1, 60%	complement f. B, AGT^3^, CD14, FLJ20244, CDKN1A	TNF-b', C4BP b', ARHGDIB, CTNNA1, C1S, SERPINB6	MCP, BLCAP, DEK, DUT	‡ DDH hepatic, PGK1, PTTG1	SAP18, PRSS1,	CG/LH/FSH/TSH a', ALDH1A1, LOC51231, PWP1, EIF3S6, TARS, DC6, GGPS1

Prediction for potential targets of E2F, IRF1, ISGF3, HNF4, PPAR, SF-1 and STAT1 were done by combined PWM & SiteGA approach. For each TFs stringencies for SiteGA and PWM are in terms of the same fixed TP rate (50–80%). Known targets – experimentally confirmed targets; very possible or possible targets – a lot of or some indirect evidence on the target functionality; BSs of homologous TFs – potential targets of homologous TFs approved or may be supposed; possibly unrelated – presumably false positives, unknown – no information on the target functionality. ^2 ^– gene have two predicted sites; ^3 ^– gene have three predicted sites; † – potential targets of members of IRF family; # – potential targets of LRH-1, that is homolog of SF-1; ‡ – potential targets of members of STAT family.

### Analysis of whole human genome

**Figure 8:**
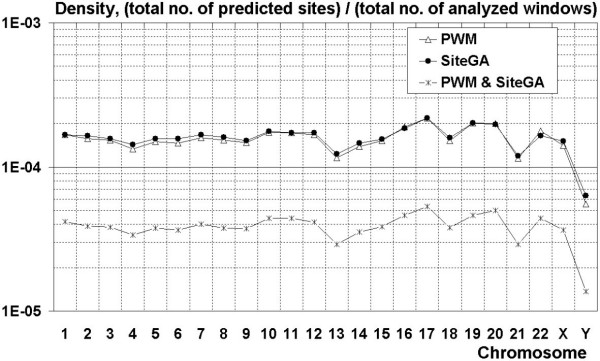
**Comparative analysis of predicted SF1 BSs densities for human chromosomes**. X-axis – number of human chromosomes (1–22, X, Y); Y axis (logarithmic scale) – ratio of no. of predicted sites to the total no. of analyzed window positions.

Finally to estimate a FP rate on a real genome sequence we evaluate potential SF1 site density for whole human chromosomes (Figure 8). The stringency was the same as for EPD analysis, i.e. TP rate fixed at 60% for SiteGA and optimized PWM). Obviously, whole genome sequences have a very small portion of functional sites, even if some sequences may bind TF in vitro, the did not bind TF in vivo. There are several alternative explanations for this (see Discussion section below).

### SiteGA web tool

**Figure 9:**
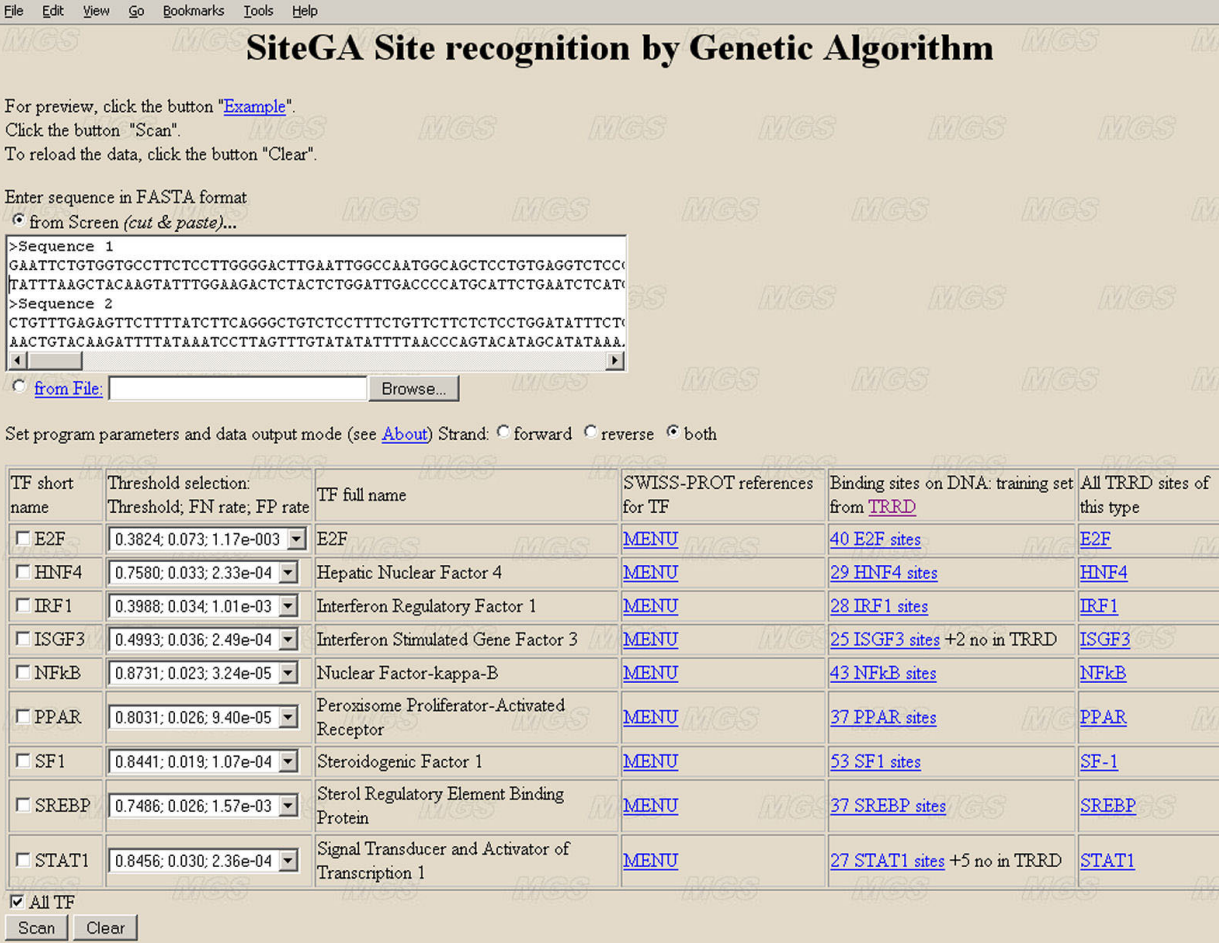
Web interface of the SiteGA tool.

**Figure 10:**
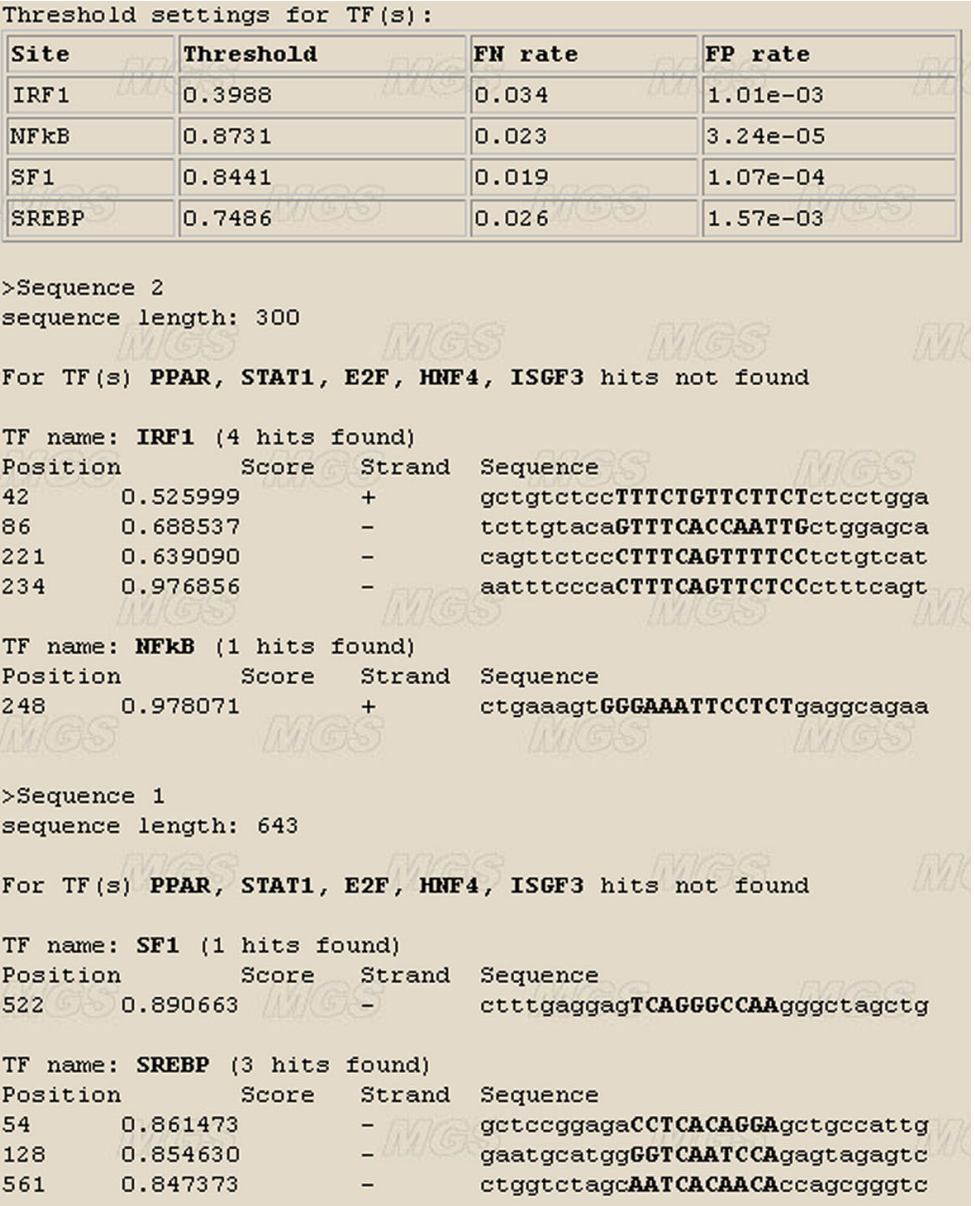
Example of the SiteGA tool output data.

The SiteGA web tool [31] has undergone major revision and rationalisation since it was first reported [33]. It now allows the user to select a subset of recognition methods, so that the output provides results for every TFBS in turn. The input data for SiteGA (Figure 9, the sequence box) should be in FASTA format. The web interface allows the user to search for 9 TFBSs. The tool starts when the TF, strand and thresholds options are specified. All threshold settings are supplied with corresponding FN and FP rates. The interface also provides TF full names and links to the SWISSPROT data, the training-set sites and all sites in TRRD [42]. An example output data is given in Figure 10. First of all, the table denotes threshold settings for all predicted sites, and then sites are listed for which no hits were found. The results for each sequence and predicted site in turn are printed, specifying sequence name and total length, then TF type, total hit count and the list of predicted sites sorted by position order. For each predicted site, the output includes its score, strand and short sequence containing the most conservative region (10–15 nt) in bold uppercase. Adding new TFBSs as sufficient functional site sequences become available is constantly refining the web tool.

## Discussion

We propose a supplementary approach for TFBS prediction, entitled SiteGA. It is based on the detection of locally positioned dinucleotides, identified from known sites using a GA with discriminant analysis. We have previously applied this combination for computer analysis of nucleosome formation potentials, RECON [43,44], which has been extensively validated and used to investigate a variety of genomic locations [45-50]. The approach has also proved for the *Drosophila melanogaster *promoter recognition [32]. The techniques for modelling dependencies between distant positions has been successfully used for the recognition of splicing sites [51-53], recombination sites [54] and genes [55].

Recently [33], we introduced the SiteGA method as one among other alternatives to traditional PWM approach. Now we propose a far more comprehensive study of TFBS recognition, which includes a range of modifications/improvements to the SiteGA method and web tool itself: (a) one window system for training instead three-window system was used (in [33] – whole window was divided into three overlapping regions of equal length of 25–35 nt (center, left flank, right flank); then, a set of locally positioned dinucleotides was sought for each of the regions separately, finally three recognition function combined); (b) three new types of recombination in genetic algorithm were applied and described (section Method, SiteGA, genetic algorithm), in [33] only one operator was used and described. Moreover, in comparison with the previous paper [33], the method is more carefully presented here, with more details of the implementation and algorithm. The careful comparison included jackknife (leave-one-out cross-validations) and bootstrap tests for accuracy estimation. These tests were done for all the SiteGA and optimized PWMs recognition models (9 TFBSs). Among other conclusion from these tests, we demonstrated that PWMs may be significantly improved by:

a) dinucleotide statistics (in contrast to mononucleotide statistics, that usually applied; and

b) exhaustive search among different length and location of PWM window.

Chen *et al*. [53] have studied selecting a window size for the acceptor and donor splice site sequence. They suggested an optimal length for the donor and acceptor splice site, i.e. a window from 9 bases upstream to 9 bases downstream for the donor splice site, and a window from 27 bases upstream to 9 bases downstream of acceptor splice site. Thus, a proper window size is among the most important factors for performance improvement.

A similar effect of window size (motif width) on the accuracy was investigated in the comparison of five motif discovery algorithms by Hu *et al*. [56]. From this comparison we may conclude that very short motif width showed the worst results. Finally, they suggested running algorithms multiple times with different motif widths to get the best result. Thus, we followed this advice and performed exhaustive searches of window size for all 9 TFBSs. Though, we should notice that motif discovery is not the same as site recognition, but intrinsically the approaches have many similar features.

We applied the algorithms of Berg and von Hippel's [2], MATCH [57] and log-odds [4,58], as well as our own variant of the latter and compared these. For many types of TFBSs, we have shown that our matrices perform better (see Figure 1, Table 1, Table 2)

Nine TFBSs have been investigated in this study. E2F is a key regulator of cell cycle. The good recognition performance achieved for this TF (Figure 3i) may be considered as a consequence of it participation in composite elements and a number of additional context and structural features in the flanking regions [18]. TFs ISGF3 and STAT1 are strongly inducible TFs [59]. ISGF3 is activated by interferons type I. STAT1 may be activated by all interferons and some other cytokines. The ISGF3 and STAT1 enhance transcription of many interferon-inducible genes at early stage of induction (1–2 hours), whereas IRF1 ensures enhanced transcription of many interferon-inducible genes for a long time in infected cells [60,61]. SF-1 is a key regulator of the steroidogenic genes expression in gonads and adrenals [62]. Moreover, SF-1 is required for development and differentiation at all the levels of the hypothalamic-pituitary-gonadal and adrenal axis [62]. There is experimental evidence for the presence of the SF-1 BSs in the regulatory regions of genes functioning within this axis [40]. NF-κB is a factor involved in regulation of many types of genes, being induced by cytokines, growth factors, and some other stimuli. NF-κB is involved not only in regulation of the immune response, but also of many other processes [41]. Nevertheless, the significance of interactions between distant positions and their competence for recognition improvement was already confirmed for NF-κB BSs [63]. The BSs of PPAR were already found earlier as good examples of interactions between distant positions [22]. Markov model application for large HNF4α set (71 sites) revealed many dependent non-adjacent positions [64]. Also this discovery confirmed the importance of a large dataset for performance improvement.

There is ample evidence to suggest that the duplex-DNA quaternary structures in the TF-DNA complex and site flanking regions are the main factors that explain the observed differences between accuracy estimates of traditional PWMs and other models which consider distant interactions. Taking into account the relations between TFBSs recognition accuracy, potential involvement of TFs in specific regulation or in processes in wide range of systems and tissues we may suppose that accuracy estimates reflected the hidden context pattern of TFBSs. Most probably duplex-DNA quaternary structures, which we have here interpreted as a set of mutually adjusted locally positioned dinucleotides, may be more strictly predefined if we find a stronger pattern of these context features.

In the current work, the sophisticated method SiteGA was developed for TFBS recognition. In order to evaluate the efficiency of our approach, we developed special techniques for traditional PWMs optimization. Namely, the best of mononucleotide or dinucleotide PWMs and optimal window lengths chosen using jackknife tests were implemented for 9 types of TFBSs. We revealed that for six TFBSs (E2F, ISGF3, IRF1, NF-κB, PPAR and SF-1) performances are better and optimal lengths are longer (Figure 2) than for HNF4, SREBP and STAT1. Maybe each of those six TFBSs has a stronger context pattern or they have a more stable set of general co-factors. The latter case may be for example if a quantity of genes, subjected to TF-specific regulation may be roughly functionally restricted.

In comparison with other well-known approaches for weights calculation [2,4,61,62], the new formula developed here (equation (1d), section Method, PWMs) performed on a par if not better than the best of the others. We further optimized our PWMs through the adjustment of lengths by jackknife tests, which were mainly based on dinucleotide statistics (Table 3). Thus we have captured the longest lengths (17–38 nt, Table 3) than traditional widely used PWMs (~10–15 nt) [2,4,58] and our PWMs have shown very substantial performance improvement (Figure 1).

Performance estimates for all PWM models did not notably depend on the exact type of resampling tests (Figure 3, jackknife or bootstrap). The same was observed in almost all cases for SiteGA models. The possible exclusions are SiteGA models for IRF1 and PPAR BSs (Figure 3g and 3d). At least for the former, this may be related with small dataset size (28, Table 3). For the latter, this effect is not so notable. In all other cases the differences between jackknife or bootstrap tests for SiteGA models were not observed. Additional sites for training cause the differences between jackknife and bootstrap tests, i.e. this may be interpreted not only as a result of substantially small, but rather not sufficient data. SiteGA algorithm in contrast to PWM's is essentially stochastic, since SiteGA as all other GAs do not guarantee the best solution. Since SiteGA accuracies did not notably depend on the type of resampling tests we may conclude that we achieved sufficient stability for SiteGA algorithm convergence.

Generally, based on 9 types of TFBSs, optimized PWM and SiteGA have shown similar performances (Figure 3). By taking into account fuzzy local positioning of the dinucleotide context, one can possibly achieve considerable increase in the recognition accuracy when compared to that for PWMs. Recall that PWM cannot be quite correct since it based on the assumption of additive contribution of site positions to the total score [5-8,20-25]. Thereby account of dependant contributions of different positions may help to improve the recognition accuracy.

For E2F, PPAR, NF-κB, HNF4 BSs we found other evidence for distant structural interactions [18,22,63,64]. For first three of them SiteGA successfully competed with performance of PWMs (Figure 3i,d,e). The worse results for HNF4 (Figure 3a) may be related to a small dataset size (we used only 29 BSs, against 71 declared in [64]), so even with PWMs we were able to catch the shortest length (Figure 2). Also we found SF-1 as a good example in SiteGA comparison with PWMs (Figure 3f).

Worse results achieved for other TFBSs can probably be attributed to two reasons. The first is a small and heterogenous dataset (this refers to ISGF3 and IRF1, Figure 3g and 3h). We may suspect that in terms of distant interactions these sets were not representative. The solution space of any complicated model (like SiteGA) is incommensurably greater than that for simple (PWM) models. Hence only a statistically large enough dataset size may allow a complicated model to realise its potential advantages. Whatever the case, the representative data are the result of a compromise between their heterogeneity and their total amount. The ISGF3 and IRF1 are very divergent among other training sets: firstly they are the smallest sets, secondly the total count of dinucleotides exploited by their SiteGA models are very small in comparison with all other SiteGA models (Table 3, last column).

The second reason for worse accuracy of SiteGA in comparison with PWMs is a weak and "short" context pattern (SREBP and STAT1, Figure 3b and 3c), since these TFBSs are found among the worst among all PWM models, and among the others they have nearly shortest lengths (Figure 2). One may expect that for some TFBSs, distant interactions may be substantially less important than close ones, so that additivity assumption [8] supported by observed context pattern.

Comparisons between the performance of PWM and SiteGA models estimated by resampling tests (Figure 3) lead us to conclude that PWMs perform better when only smaller datasets are available (ISGF3 and IRF1, Table 3). SiteGA models need more representative training sets (> 25–30 sites) to achieve better results. The comparison of performances for datasets of different size suggests that a subtle context pattern, which may increase the performance, might be successfully retrieved only from larger training data [10,25].

Simulated data analyses have shown that performances of mono- and dinucleotide PWMs, as well as of optimized markov model (OMiM, [25]) are increased when dataset size grows from 15 to 150 sequences. Moreover, the most substantial growth of performance for OMiM was observed when the dataset size was increased from 15 up to 75 sites. This observation allows us to suppose that nearly all our datasets (Table 3) still far from the optimal size.

We realise, that as a quite sophisticated model, SiteGA in comparison with PWMs may be prone to overfitting. In the case of overfitting, the accuracy achieved by the training data should be quite good, but the independent control data do not confirm good performance. This effect should be noticeable for a relatively small dataset and careful resampling tests allow us to appreciate possible overfitting effects. In our paper, we carried out two types of resampling tests (jackknife and bootstrap), thus, the crucial interference of overfitting is excluded.

To conclude, we may note that increasing the number of training sequences makes a sophisticated method like SiteGA less prone to overfitting, thus in the case of larger datasets SiteGA may successfully compete with optimized PWMs.

We may emphasize at least three possible ways for further improvement of SiteGA:

(1) Extension of all current TFBSs datasets up to at least ~40–60 sequences;

(2) Careful adjustment of SiteGA-specific parameters (e.g. number of LPDs);

(3) Analysis of different lengths. That was extremely important for PWMs (Figure 2), but still SiteGA models have been implemented with predefined lengths. But there is no guarantee that PWMs and SiteGA have exactly the same setting for optimal window length.

Next our task was to study the hidden context patterns that allow better recognition performance. For example, the SF-1 BS has been described by the motifs CCAAGGTCA [65], (C/T)CAAGGT(C/T)A [66] and GTCAAGGTCA, that was derived from our data set [40]. According to the data extracted from TRRD [42], the region protected from nuclease digestion in the footprinting experiment was not longer than 20 bp. It followed that the core region of the SF-1 BS was not longer than that. We took local dinucleotides of the SiteGA SF-1 model and studied the distribution of their locations within and outside the core region. We found that most significant SF-1 context features were inherent to the consensus [10,19] and footprint (approximately [5,24]) regions (Figure 4).

The revealed dependencies between locally positioned dinucleotides for all SiteGA models were split into close and distant ones (Figure 5), which revealed that:

(1) The most significant correlations are mainly between pairs of close dinucleotides, mostly resided to the core region (most probably these patterns are clearly handled by PWMs);

(2) Larger portions of less significant correlations are mainly between distant dinucleotides;

(3) Total numbers of distant dependencies are substantially higher than numbers of close ones. Since the significance of the distant dependencies is generally lower than for the close ones, the larger dataset is favourable for detailed clarification. We may note that the domination of close dependencies among most significant correlations (Figure 4, p < 0.001) is indeed the basis for assumption of independence [8], which was accepted in our case by PWMs.

Finally, a large portion of the current research was devoted to analysis of EPD data, i.e. nearly all human promoters ~10% of genome (genes for which the transcription start site has been determined experimentally). This is a substantial development that is worth bringing to the attention of a wider audience. Firstly we separately used optimized PWM and SiteGA models on human EPD promoters (Figure 6) and simulated data analysis (Figure 7), and applied stringent thresholds (TP 50%–70%) to reduce the number of FPs. A small number of predicted sites may be considered as indirect evidence of better recognition performance, since we can expect obvious enrichment of total EPD dataset with functional TFBSs. Indeed for both PWMs and SiteGA, the ratios of predicted site frequencies to those for background (random sequences, Markov model 0) varied from ~1 to ~3 at the most permissive stringency (TP 70% for SiteGA and PWMs) depending on TF type.

The comparison of PWM and SiteGA model predictions applied to EPD promoter data (Figure 6) as well as for random sets (Figure 7) suggests the following:

(1) For E2F, HNF4, NF-κB, SF-1 and STAT1, SiteGA appeared to be better than the respective PWMs. Some deviation from this general rule is not very important. For instance, a small advantage of E2F PWM at TP 50% (Figure 6) was suppressed by considerable dominance of SiteGA at two other thresholds, TP 60% and 70%.

(2) Ambiguous situations are found for PPAR and SREBP: the ratios of PWM and SiteGA predictions (Figure 6, 7) may be more or less than unity depending on threshold, but generally it is hard to choose the better model.

(3) For IRF1 and ISGF3, PWMs achieved better results than SiteGA, which may be attributed to their having only small datasets. Another possible explanation is the small total count of LPDs captured by SiteGA models (38 and 36 are two most divergent values in the last column of Table 3).

This EPD analysis suggests that SiteGA models are able to outperform the optimized dinucleotide PWMs.

Table 4 shows the genes (identified by the TFBS searches) sorted on the likelihood (from biological knowledge) of them genuinely regulated by the TF concerned. For all TFBSs, the superposition of PWM and SiteGA appeared to reduce FPs substantially, while TPs reduced slower (Figure 6). We have shown that among combined predictions indeed there are many known and other probable functional sites (Table 4). The combined use of both models is very promising for whole genome searches, since the human genome contains approximately tenfold greater number of genes than is present in EPD now.

Analysis of whole human genome sequences (Figure 8) confirmed that SiteGA and optimized PWMs have similar performances and the combination of two different approaches has a substantially reduced FP rate. Obviously, the density of predicted sites for Y chromosome is an outlier among others, most probably explained by differences in base content and gene density [67-69]. The average predicted site density for other chromosomes (1–22, X) are 1.60E-04, 1.64E-04 and 3.99E-05 for optimized PWM, SiteGA and their combination, respectively (Figure 8). These figures are slightly lower than the random sequence scores for PWMs and SiteGA of 2.0E-04 (see Figure 3f), which is probably accounted for by minor differences in base composition. Whereas for EPD promoters, there were notably higher predicted-site densities (2.18E-04, 2.19E-04 and 5.56E-05, respectively). This may be just a consequence of closer similarity of nucleotide contents between training data and EPD promoters (they are both gene regulatory regions), than between training data and full-length chromosomes.

Finally, we consider the high number of predicted SF-1 BSs on human chromosomes (Figure 8). The first and obvious explanation is the presence of BSs that have similar consensus sequences. For instance, SF-1 BSs closely resemble those of LRH-1 [70], both TFs function as monomers. In general, closely related TFs share the same consensus (e.g. androgen, progesterone and glucocorticoid receptors that bind DNA as dimers [71,72]). Futhermore, the specific location of an predicted site may be non-functional *in vivo *for a range of reasons: (a) the position may lie in tightly packaged heterochromatin far from gene regulatory machinery (b) although in putatively regulatory DNA, it also may be not exposed enough for TF binding, and finally (c) in a given tissue or development stage where the TF is present, only a subset of the potential binding sites are available. Hence in genomic DNA, we may separate '*in vitro' *and '*in vivo' *false positives. That means that the latter can bind TF *in vitro*, but *in vivo *this interaction is not observed. We have previously confirmed that our computer tools are able to predict functional sites at least *in vitro *quite well [73]. However, the majority of predicted sites on human chromosomes are probably '*in vivo' *false positives, which can only filtered out with extra knowledge.

## Conclusion

We have refined the SiteGA approach for TFBS prediction. The approach uses a genetic algorithm with a discriminant function of locally positioned dinucleotides (LPDs) to find out the most important positions and dinucleotides. This technique provides a mechanism to infer and apply long-range structural TF-DNA interactions. SiteGA and optimized PWMs have been applied to 9 TFBSs (E2F, HNF4, ISGF3, IRF1, NF-κB, PPAR, SF-1, SREBP, STAT1). We performed jackknife and bootstrap resampling tests to compare performances of SiteGA and PWMs, than we applied both methods separately to EPD promoter data. These comparisons allow us to conclude that for the SiteGA models and optimized PWMs have similar performances. The analysis of dependencies between frequencies of LPDs found by SiteGA models revealed a number of significant correlations between close and distant LPDs. Analysis has shown that the majority among the most significant of these correlations are close and mainly located in the core (footprint) region. Among the less significant correlations, the distant were dominating and resided in both core and flanking regions. Finally we applied combined SiteGA & PWM approach to EPD promoter data. We have demonstrated that the combined approach effectively reduced the false positive rate, which is especially important for higher eukaryotes, whose regulatory regions are long and poorly annotated. This combination looks very promising for future genome-wide searches, since the two different models together ensured a substantial reduction in the number of false positives. Thereby only the most reliable potential TF targets may be found. The SiteGA web tool interface [31] for 9 TFBSs types has been implemented.

## Methods

### Sequence data

The TFBS training samples of E2F, IRF1, ISGF3, HNF4, NF-κB, PPAR, SF-1, SREBP and STAT1 (Table 3) were derived from the TRRD [42] and contained only experimentally confirmed sites (see also presentation of all training samples on the web tool homepage, [31]). Only TFs with at least 25 BSs were considered. Initially the analyzed regions were retrieved as a 40–50 base windows. The conserved sequence motifs are at the centre of these windows. The sequence lengths used for analysis and comparison of PWMs and SiteGA were adjusted by preliminary jackknife tests performed for PWMs (see section Resampling tests below).

In order to preserve the validity of the resampling tests, we removed duplicate sequences from each BS set. Additionally sequence similarity was checked for all sets to check for homologous genes. Very few similar sequences were found. The application of a 90% similarity threshold revealed two pairs of homologous sequences in IRF1, PPAR and NF-κB sets, none in the SREBP set, and all the remaining sets had one pair of homologous sequences. The majority of BSs were represented by nonorthologous sequences. Thus, among 53 distinct sites comprising the largest SF-1 set, 38 (~72%) were in unique non-orthologous vertebrate genes and the remaining 15 belonging to 7 groups of orthologous genes.

For the EPD searches [38], 1871 sequences of lengths 600 nt located [-550;+50] relative to transcription start sites were used.

Full-length sequences of human chromosomes were downloaded from the NCBI site [74].

### PWMs

Mononucleotide and dinucleotide PWMs (mono- and diPWMs) were calculated on the basis of nucleotide and dinucleotide frequencies respectively, i.e. for monoPWMs {*S*_*j*_} = {A, T, G, C}, *j *= *1*,.., *J *= 4 and for diPWM {*S*_*j*_} = {AA, AT,..., CC}, *j *= 1,.., *J *= *16*. Let *n*_*i*,*j *_denote the number of sites for which (di-)nucleotide *S*_*j *_appears in position *i*, and *p*_*j *_is background (expected) frequency of (di-)nucleotide *j*. We investigated four approaches for weight computation (Eqs 1a-1d):

(1a) $$w_{i,j}^{\left(BVH\right)}=Ln\left[\frac{n_{i,j}+0.5}{n_{i,\max}+0.5}\right],$$

Berg and von Hippel approach (BVH) [2],

*n*_*i*,max _– the number of occurrences of the most common (di-)nucleotide in position *j *of the set of binding sites;

(1b) $$w_{i,j}^{\left(LOD\right)}=Ln\left[\frac{n_{i,j}+b_{j}}{N+b}/p_{j}\right],$$

log-odds approach (LOD) [4,58].

*b*_*j *_= *I*/*J*, $b=\sum_{j=1}^{J}b_{j}=1$, pseudocounts settings, various pseudocounts settings are used in PWMs, [21], *N *– the number of BSs in the set;

(1c) $$w_{i,j}^{\left(MCH\right)}=n_{i,j}\times \sum_{k=1}^{J}\left[n_{i,k}\times Ln[J\times \frac{n_{i,k}}{N}]\right],$$

MATCH approach (MCH) [57],

Finally, in this work, we introduce the use of natural logarithms (NLG):

(1d) $$w_{i,j}^{\left(NLG\right)}=-n_{i,j}\times Ln[p_{j}].$$

For all approaches and a nucleotide sequence *X *[*A*,*B*] the PWM score was calculated as follows:

(2) $$\varphi_{PWM}(X)=\sum_{i=A}^{B,B-1}\sum_{j}\frac{w_{i,j}\times \delta_{i,j}(X)-w_{i,\min}}{w_{i,\max}-w_{i,\min}}$$

The indicator *δ*_*i*,*j*_(*X*) = 1, if *S*_*j *_= *X*_*i *_(nucleotide (dinucleotide) *j *occurs at *i*-th position of sequence *X*) and *δ*_*i*,*j*_(*X*) = 0 otherwise; *w*_*i*,min _and *w*_*i*,max _are minimal and maximal weights for *i*-th position. For mononucleotide and dinucleotide PWMs, the sum over *i *is calculated in the intervals [*A*,*B*] and [*A*,*B-1*] respectively. The PWM score is restricted to the interval [0; 1].

### PWM optimization

NLG PWMs were optimized as follows. The training window size and location search implied the search among different length (from 10 to 40–50 nt) and locations, i.e. for each window size three slightly shifted (1 nt) locations were tested. We chose for each TFBS type the best matrix type (mono- or dinucleotide PWMs). The recognition accuracy estimates based on the jackknife resampling tests [39] were used for window size, location and matrix type selection. We compared PWMs models, based on optimized settings for NLG approach with other PWMs: monoPWM (Table 1) and diPWM (Table 2), lengths 15 nt and 20 nt, correspondingly). For weights calculation BVH, LOD, MCH and NLG approaches were used.

### SiteGA, genetic algorithm

The SiteGA method employed a discriminant function of locally positioned dinucleotides (LPDs). Identification of these LPDs was directed by a genetic algorithm (GA), which handled a population of individuals. An individual was represented as a set of *N *LPD frequencies. Each LPD Λ(*s*, *e*, *d*) was defined by location [*s*, *e*] within the whole window [*A*,*B*] and dinucleotide type *d *(*d*-type), 1 ≤ *d *≤ 16. The start *s *and the end *e *denote limits for possible positions of first base of the dinucleotide, so that *A *≤ *s *≤ *e *≤ *B-1*. The initial GA population consisted of individuals of arbitrarily assigned LPDs. Note that for any individual overlapping of locations for two LPDs of the same dinucleotide type is forbidden. After the population initiation GA produced iterative mutations and recombinations. Two types of mutations were applied. Consider an individual and fix an LPD Λ(*s*_*n*_, *e*_*n*_, *d*_*n*_), 1 ≤ *n *≤ *N*. The mutation implied either location [*s*_*n*_, *e*_*n*_] or dinucleotide type *d*_*n *_change. Recombinations were defined in a more complicated fashion. Generally recombination between two parent individuals means an exchange of two or more different LPDs or their "parts". First, we take two distinct individuals and consider one of them. Then, we sort all LPDs in ascending order of dinucleotide types and starts. Next we denote $\sigma (d)=\underset{1\leq k\leq N}{Min}(s_{k},d_{k}=d)$, $\varepsilon (d)=\underset{1\leq k\leq N}{Max}(e_{k},d_{k}=d)$, and two respective order numbers are *n*(*σ*(*d*)) and *n*(*ε*(*d*)). Then for any two LPDs *n *and *n *+ 1 that are adjacent in the ordered list, we get the following: (1) if *d*_*n *_= *d*_*n *+ 1_, then *e*_*n *_<*s*_*n *+ 1_, otherwise (2) *d*_*n *_<*d*_*n *+ 1 _means that *e*_*n *_= *ε*(*d*_*n*_) and *s*_*n *+ 1 _= *σ*(*d*_*n *+ 1_)) correspondingly. Let *d*-type occupancy *g*_*d *_denote the total number of LPDs of *d*-type. According to previous notations, *g*_*d *_= 1 + *n*(*ε*(*d*)) - *n*(*σ*(*d*)) and summation of all occupancies gives the total number of LPDs: $\sum_{d=1}^{16}g_{d}=N$. Below the superscript denotes the 1^*st *^or 2^*nd *^individual. Next we enter notations *r1 *&*r2 *for the ordered numbers of recombinating LPDs of 1^*st *^and 2^*nd *^individuals, respectively. This defines the following types of recombination:

(1) If we have $g_{d}^{1}=g_{d}^{2}$, then all LPDs of *d*-type are exchanged: *n*(*σ*^1^(*d*)) ≤ *r1 *≤ *n*(*ε*^1^(*d*)) &*n*(*σ*^2^(*d*)) ≤ *r2 *≤ *n*(*ε*^2^(*d*));

(2) If for two LPDs $\Lambda (s_{n1}^{1},e_{n1}^{1},d)$ &$\Lambda (s_{n2}^{2},e_{n2}^{2},d)$ a position *x *(*A *<*x *<*B-1*) is chosen so that $s_{n1}^{1}\leq x\leq e_{n1}^{1}$ &$s_{n2}^{2}\leq x\leq e_{n2}^{2}$ respectively, then these LPDs are exchanged: *r1 *= *n1 *&*r2 *= *n2*;

(3) We again need the same as (2) above and yet $g_{d}^{1}=g_{d}^{2}$, *n1-n*(*σ*^1^(*d*)) = *n2-n*(*σ*^2^(*d*)). LPDs of *d*-type whose starts and ends are larger than *x *are exchanged: *n*1 <*r*1 ≤ *n*(*ε*^1^(*d*)) &*n*2 <*r*2 ≤ *n*(*ε*^2^(*d*)), and for two LPDs only the ends are exchanged: *r1 *= *n1 *&*r2 *= *n2*;

(4) If for two LPDs $\Lambda (s_{n1}^{1},e_{n1}^{1},d_{n1}^{1})$ &$\Lambda (s_{n2}^{2},e_{n2}^{2},d_{n2}^{2})$ and two other pairs of adjacent in the ordered lists LPDs $\left\{\Lambda (s_{k2}^{2},e_{k2}^{2},d_{k2}^{2}),\Lambda (s_{k2+1}^{2},e_{k2+1}^{2},d_{k2+1}^{2})\right\}$ &$\left\{\Lambda (s_{k1}^{1},e_{k1}^{1},d_{k1}^{1}),\Lambda (s_{k1+1}^{1},e_{k1+1}^{1},d_{k1+1}^{1})\right\}$, we have: {$\left\{s_{n1}^{1}>e_{k2}^{2}\text{ or }d_{n1}^{1}>d_{k2}^{2}\right\}$ and $\left\{e_{n1}^{1}>s_{k2+1}^{2}\text{ or }d_{n1}^{1}>d_{k2+1}^{2}\right\}$} & {$\left\{s_{n2}^{2}>e_{k1}^{1}\text{ or }d_{n2}^{2}>d_{k1}^{1}\right\}$ and $\left\{e_{n2}^{2}<s_{k1+1}^{1}\text{ or }d_{n2}^{1}<d_{k1+1}^{2}\right\}$}, then two original LPDs are exchanged: *r1 *= *n1 *&*r2 *= *n2*.

### SiteGA, discriminant analysis

The GA process was based on the fitness function maximization. The fitness evaluated each individual of population. Let us consider the real (1^st^) and random (2^nd^) sequence sets. Random sequences were obtained by shuffling the nucleotides within real sequences (Markov model 0). The fitness was given by the Mahalanobis distance *R*^2 ^between two sets in the space of *N *LPD frequencies:

(3) $$R^{2}=\sum_{k=1}^{N}\sum_{n=1}^{N}\{[f_{n}^{\left(1\right)}-f_{n}^{\left(2\right)}]\times S_{n,k}^{-1}\times [f_{k}^{\left(1\right)}-f_{k}^{\left(2\right)}]\}$$

Here, $f_{n}^{\left(1\right)}$ is the mean frequency of the *n*^*th *^LPD calculated for the real set; $f_{n}^{\left(2\right)}$ – respective frequency for the random set; and $S_{n,k}^{-1}$ is an element of the matrix |*S*^-1^| inverse to the matrix |*S*| = |*S*^(1)^| + |*S*^(2)^|. These are the covariance matrices of the vectors of LPDs over the 1^st ^and 2^nd ^sets, correspondingly. In our implementation, only individuals with equal numbers of LPDs (*N*) were compared by the fitness *R*^2^. The choice of the suitable *N *value was based on the resampling tests (see below section Resampling tests). Among others GA parameters, the population size may be noted as the most important. It was adjusted on the basis of a trade-off between calculation time and algorithm convergence.

After the GA finished the final set {*f*_*n*_(*X*)} of *N *LPDs, frequencies may be attributed to any nucleotide sequence *X *[*A*,*B*]. Then we consider two additional designations:

(4) βn=1R2×∑k=1N{Sn,k−1×[fk(1)−fk(2)]},γ=−(12)×∑n=1N{βn×[fn(1)+fn(2)]}

This allows deduction of the SiteGA score as follows:

(5) $$\varphi_{SiteGA}(X)=1-|1-(\sum_{n=1}^{N}\{\beta_{n}\times f_{n}(X)\}+\gamma )|$$

The highest score +1 denotes the best prediction. Zero score implies an arbitrary classification between real and random sets.

### Resampling tests

Resampling tests were performed for all 9 TFBSs (Table 3) to compare the performance of PWM and SiteGA models. The random nucleotide sequences were included in background sets. For each TFBS type, we applied resampling jackknife (leave-one-out cross-validations) and bootstrap tests [39]. The jackknife test sampled data by taking out a single site, then training a model and using the omitted site for validation. This process was repeated for every site in the full set. Bootstrapping differed from jackknifing in that rather than taking only one site out, it randomly selected 90% of the full set for training, while the remaining sites were used for validation. A total of 100 bootstrap iterations were done.

Both types of resampling tests were applied to the same data, in order to verify the stability of performance estimates and to study the relationship between sample size and performance.

Note that we used as background models for SiteGA and optimized PWMs the 0^th ^order Markov models, which have been widely used for performance estimates [15,23], since this type of sequences is lack any significant dependencies among different positions, but still preserves the nucleotide content of training foreground data. Though there are many attempts to use genomic sequences as a background model or a contrast negative set (e.g. second [57] or third [75] exons, random promoters [22], random position in the genome [76]), contamination of such negative sets with functional (at least *in vitro*) binding sites is possible. Thus, we used as a background set random, 0^th ^order Markov model sequences, which surely left only a small chance to functional site formation.

### Performance measures

The comparison of PWM and SiteGA models was based on the computation of the ROC plot, i.e. dependence between true positive (TP) and false positive (FP) rates. As the main performance measure, FP rates were chosen which corresponded to TP rates in the range 50%–70%. This range was accepted since it primarily related to stringent thresholds applied in wide genome analysis. For example, the most stringent threshold (TP rate 50%) corresponds to the loss of approximately a half of potential sites, at the same time the FP rate is very efficiently suppressed. Usually genome data require minimization of FP rates; therefore the most stringent thresholds have applied in this work.

### PWM and SiteGA models settings

The details for developed PWM and SiteGA models for TFBSs of 9 types are shown in Table 3. The performance of PWM model strongly depends on the window length. Another parameter of PWM model is the matrix type, since we chose between mononucleotide and dinucleotide matrices. The jackknife tests were used for selection of window length and location. The window length and location selection implied the search among different sizes (from 10 to 40–50 nt) and locations, i.e. for each window length three overlapped and slightly shifted locations (1 nt) were tested. The background set generated by shuffling of control site sequence (taken in it full length 40–50 nt). To build SiteGA models, we chose for all TFs the same BS lengths as for corresponding PWM models (Table 3). The total number of LPDs was adapted by SiteGA model using resampling tests by choosing the value that maximized the performance.

### Combination PWM & SiteGA

The combination PWM & SiteGA implied that a potential site was predicted separately by optimized PWM and SiteGA models at specified stringencies. In our implementation we fixed TP rates for both models, thus, a fixed stringency means the recognition of certain portion of train data. For example, if for optimized PWM and SiteGA we set stringencies corresponded to TP rates 70%, then effective TP rate for combined approach will be approximately 50%. Thus, combination allows reducing the FP rates, but this also related with a moderate low of TP rate.

## Availability and requirements

Project name: SiteGA

Project home page: 

Operating system(s): Platform independent (web-based, tested on Mozilla Firefox 2.0 and Internet Explorer 6.0)

Programming language: The recognition algorithms are implemented in C++, the interactive version was developed using Perl

Licence: Free for academic and non-profit researchers. Contact the corresponding author for commercial licensing

## List of abbreviations

BS binding site

diPWM dinucleotide PWM

FP false positive

GA genetic algorithm

LPD locally positioned dinucleotide

monoPWM mononucleotide PWM

PWM position weight matrix

ROC receiver operating characteristic

TP true positive

TF transcription-factor

TFBS transcription-factors binding site

## Authors' contributions

VGL drafted the manuscript, performed the computer analysis and prepared the manuscript.

EVI, EAA and IIT prepared the sequence data and drafted the manuscript.

TIM participated in the design of the study and drafted the manuscript.

NAK and TCH drafted the manuscript and participated in the design of the study.

All authors read and approved the final manuscript.
